# DNA methylation instability by BRAF-mediated TET silencing and lifestyle-exposure divides colon cancer pathways

**DOI:** 10.1186/s13148-019-0791-1

**Published:** 2019-12-16

**Authors:** Faiza Noreen, Taya Küng, Luigi Tornillo, Hannah Parker, Miguel Silva, Stefan Weis, Giancarlo Marra, Roland Rad, Kaspar Truninger, Primo Schär

**Affiliations:** 1 Department of Biomedicine, University of Basel, Mattenstrasse 28, CH-4058 Basel, Switzerland; 2 Swiss Institute of Bioinformatics, 4053 Basel, Switzerland; 3 Institute of Pathology, University Hospital Basel, 4056 Basel, Switzerland; 4 Institute of Molecular Cancer Research, University of Zurich, 8057 Zurich, Switzerland; 5 Department of Medicine II, Klinikum Rechts der Isar, Technische Universität München, 81675 Munich, Germany; 6 Gastroenterologie Oberaargau, CH-4900 Langenthal, Switzerland

**Keywords:** Colon cancer, DNA methylation, CIMP, *BRAF*^*V600E*^, TET, Aging

## Abstract

**Background:**

Aberrations in DNA methylation are widespread in colon cancer (CC). Understanding origin and progression of DNA methylation aberrations is essential to develop effective preventive and therapeutic strategies. Here, we aimed to dissect CC subtype-specific methylation instability to understand underlying mechanisms and functions.

**Methods:**

We have assessed genome-wide DNA methylation in the healthy normal colon mucosa (HNM), precursor lesions and CCs in a first comprehensive study to delineate epigenetic change along the process of colon carcinogenesis. Mechanistically, we used stable cell lines, genetically engineered mouse model of mutant BRAF^V600E^ and molecular biology analysis to establish the role of BRAF^V600E^-mediated-TET inhibition in CpG-island methylator phenotype (CIMP) inititation.

**Results:**

We identified two distinct patterns of CpG methylation instability, determined either by age–lifestyle (CC-neutral CpGs) or genetically (CIMP-CpGs). CC-neutral-CpGs showed age-dependent hypermethylation in HNM, all precursors, and CCs, while CIMP-CpGs showed hypermethylation specifically in sessile serrated adenomas/polyps (SSA/Ps) and CIMP-CCs. *BRAF*^*V600E*^-mutated CCs and precursors showed a significant downregulation of *TET1* and *TET2* DNA demethylases. Stable expression of *BRAF*^*V600E*^ in nonCIMP CC cells and in a genetic mouse model was sufficient to repress TET1/TET2 and initiate hypermethylation at CIMP-CpGs, reversible by *BRAF*^*V600E*^ inhibition. *BRAF*^*V600E*^-driven CIMP-CpG hypermethylation occurred at genes associated with established CC pathways, effecting functional changes otherwise achieved by genetic mutation in carcinogenesis.

**Conclusions:**

Hence, while age–lifestyle-driven hypermethylation occurs generally in colon carcinogenesis, *BRAF*^*V600E*^*-*driven hypermethylation is specific for the “serrated” pathway. This knowledge will advance the use of epigenetic biomarkers to assess subgroup-specific CC risk and disease progression.

## Background

Initiation and progression of cancer is facilitated by genetic and epigenetic instability [1]. Carcinogenesis in the colon follows two distinct pathways. The “classical” polyp to cancer model describes a progressive accumulation of genetic mutations, transforming glandular epithelial cells to form tubular adenomas (TAs), advanced adenomas, and ultimately, colon cancer (CC) [2]. The alternative, “serrated” pathway accounts for 15–30% of CC and sessile serrated adenoma/polyps (SSA/Ps) are the likely precursors [3, 4]. Carcinogenesis along this pathway is associated with the acquisition of a CpG island methylator phenotype (CIMP), characterized by widespread DNA hypermethylation in gene promoter-associated CpG islands (CGIs) [5–7]. CC can be classified in CIMP- and nonCIMP-CC, although there is no consensus with respect to the hypermethylation status unambiguously defining CIMP. CIMP- and nonCIMP-CC not only develop from distinct precursors, they also show distinct clinical and genetic features. CIMP-CC typically occur in the proximal colon of elderly females, harbor a *BRAF*^*V600E*^ mutation and often show microsatellite instability (MSI) due to silencing of the mismatch repair gene *hMLH1* [8]. *By contrast,* nonCIMP-CC show little preference in location and gender; are frequently mutated in *APC*, *KRAS,* and *TP53 genes; are* microsatellite stable but often show chromosomal instability (CIN) [9]. The heterogeneity in CC suggests that cell of origin, genetic background, and environmental exposure shape the evolution of cancers with distinct genetic and epigenetic contributions and clinical features.

The genome–environment interactions underlying the acquisition of genetic and epigenetic alterations during lifetime and CC-carcinogenesis are poorly understood. Despite the strong association between *BRAF*^*V600E*^ and CIMP-CC, a molecular mechanism underlying the formation of this cancer-subtype has not been identified. Only recently, oxidative DNA demethylases, the ten-eleven translocation protein family (TET1-3), have emerged as key players in DNA hypermethylation in cancers of various tissues [10–12]. In CC, TET1 silencing was shown to be associated with *BRAF*^*V600E*^ and with CIMP-CC and its precursors [13], but mutations in TET genes are very rare in CC [14].

In the clinical management of CC, cancer stratification based on molecular subtyping has become an essential to guide treatment decisions [15]. Recent gene expression-based CC profiling identified four consensus molecular subtypes that evolve through mainly two distinct routes, separating the “serrated” and the “classical” pathways at the precursor stage [16, 17]. However, data on the normal colonic epithelium of screening individuals are too scarce to support a clear delineation of molecular events associated with the transformation of the healthy normal mucosa (HNM) to cancers as well as to determine the contribution of genetic and epigenetic factors to cancer initiation and progression along the two separate precursor to CC pathways. A better understanding of the molecular mechanisms and signatures associated with colon carcinogenesis, from the earliest events in the HNM to invasive cancer is essential to develop effective means for early detection and prevention as well as for the CC therapy.

We have previously shown that CC-specific DNA methylation changes are readily detectable in HNM [18, 19]. The aim of this study was to determine CC subtype-specific DNA methylation signatures in females, decipher their development in HNM and CC precursors, identify mechanisms underlying cancer-associated methylation change in carcinogenesis, and assess its significance for carcinogenesis. To cover the entire spectrum of carcinogenesis and achieve high cancer-specificity, we performed genome-scale DNA methylation analysis of the HNM as a reference to derive CC-specific DNA methylation signatures and examined these in precursor lesion. This identified two groups of CpGs showing distinct hypermethylation properties, discriminating the CIMP from the nonCIMP pathway of colon carcinogenesis. Age and lifestyle exposure emerged as key factors of methylation change at CpGs showing hypermethylation in all CCs, whereas genetic deregulation of TET DNA demethylases by oncogenic BRAF^V600E^ was responsible for CIMP-cancer initiation in the colon.

## Results

### DNA methylation signatures in colon cancer

**Fig. 1:**
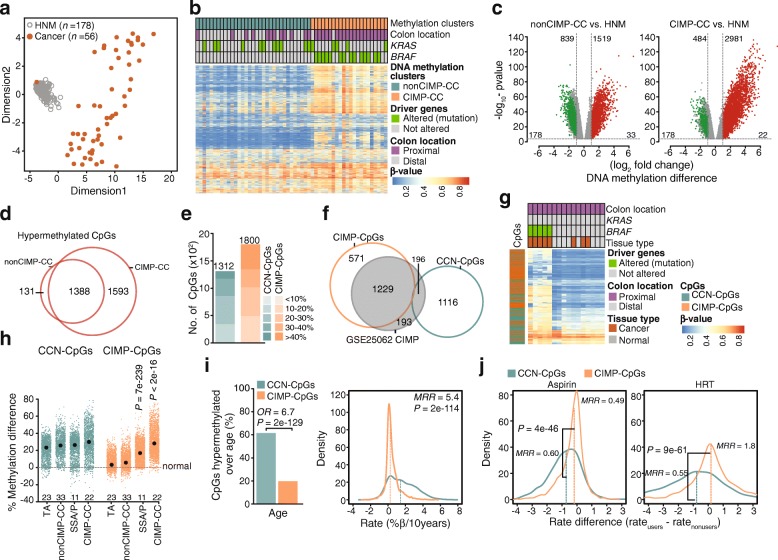
DNA methylation identifies subtype-specific hypermethylation signatures in colon cancer. **a** Genome-wide methylation profiles measured by HM27K in healthy normal colon mucosa and colon cancer. Multidimensional scaling (MDS) plot includes all probes on the array. **b** Recursively partitioned mixture model (RPMM)-based unsupervised clustering of colon cancer (*n* = 55) methylation profiles from panel *a*. Heatmap shows β-values of CpGs (5254) with SD > 0.16 across all samples (top variable probes). **c** Volcano plots show differential methylation analysis between nonCIMP- and CIMP-CCs vs. healthy normal colon mucosa (HNM). Colored dots represent significant (*P* < 0.0001, fold change > 2, β-difference > 10%) hypomethylated (green) or hypermethylated (red). At the top number of significant CpGs and at the bottom number of samples are shown. The *x*-axis denotes log_2_ fold changes in methylation relative to HNM and *y*-axis denotes −log_10_ of false discovery rate (FDR)-adjusted *P* value. **d** Venn diagrams show comparisons of hypermethylated CpGs identified in nonCIMP-CC and CIMP-CCs. **e** The number of CC-neutral-CpGs (CCN-CpGs) and CIMP-CC-specific CpGs (CIMP-CpGs). **f** Venn diagram shows comparisons of CCN-CpGs and CIMP-CpGs with previously published sites methylated in CIMP-cancers (GSE25062). **g** Heatmap shows β-values of CCN-CpGs (1312) and CIMP-CpGs (1800) in eight cancers and paired normal tissues. **h** Dot plots show methylation difference at CCN-CpGs and CIMP-CpGs between tubular adenoma (TA), sessile serrated adenoma/polyp (SSA/P), nonCIMP-CC, and CIMP-CC from paired normal tissue (dashed line). Black circles show medians. *P* values were calculated with Wilcoxon rank-sum test. **i** Bar plot shows number of CCN-CpGs and CIMP-CpGs hypermethylated over age in the HNM (*left*). *P* values and odds ratio (*OR*) were calculated with Fisher's exact test. Density plot shows the rates of methylation change per 10 years of age (*right*). *P* values were calculated with Wilcoxon rank-sum test. Methylation rate ratio (MRR); median-rate_CCN-CpGs_ / median-rate_CIMP-CpGs_. **j** Density plot shows the difference in the rate of methylation change in HNM from aspirin users (≥ 2 years) or HRT users (after age 50) to nonusers. *P* values were calculated with Wilcoxon rank-sum test. Methylation rate ratio (MRR); median-rate_user_ / median-rate_nonuser_

We restricted our analysis to the samples from females only, taken from either the proximal or the distal colon (no rectum). All published data sets used in this study were also following these criteria. To segregate DNA methylation subtypes across CCs, we analyzed publicly available Infinium HumanMethylation27K array (HM27K) data on 56 cancers [20] of the proximal and distal colon of female individuals and 178 biopsies of normal mucosa of healthy females (HNM) [19]. We based our analysis on the previous data on a cohort that included detailed lifestyle information [19], which at that time was generated on HM27K. Multidimensional scaling (MDS) showed a clear separation of cancers from the HNM, except for one cancer (Fig. 1a), which was therefore excluded from further analyses. Unsupervised hierarchical clustering of the DNA methylation data of the remaining 55 cancers identified two main clusters (Fig. 1b); cluster A contained all cancers with a wild-type BRAF (BRAF^WT^) status, most of them located in the distal colon (21/33, 63%), cluster B mainly contained BRAF^V600E^-mutated cancers (14/22, 64%) located in the proximal colon (20/22, 91%). A substantial fraction of cluster A cancers was indeed previously classified as nonCIMP (26/33, 79%) and cancers in cluster B were classified as CIMP-high (19/22, 86%) [20]. We will refer to cluster B as CIMP-CCs and to cluster A as nonCIMP-CCs.

To define CC-subtype-specific DNA methylation signatures, we compared the methylation profiles of CIMP- and nonCIMP-CCs with those of HNM [19]. This identified 1519 CpGs showing hypermethylation and 839 CpGs showing hypomethylation in nonCIMP-CCs, and 2981 CpGs showing hyper- and 484 showing hypomethylation in CIMP-CCs (Fig. 1c). Because of the well-established role of DNA hypermethylation in CC biology, we focused further analyses on the hypermethylated CpGs. Amongst all hypermethylated CpGs, 131 were specific for nonCIMP-CC, 1593 for CIMP-CCs, and 1388 were common to both cancer-subtypes (Fig. 1d). Yet, 207 of these commonly hypermethylated CpGs showed significantly higher methylation levels in CIMP-CCs than in nonCIMP-CCs. Given this, we defined two classes of hypermethylated CpGs in cancer: (i) CIMP-CC-specific CpGs (CIMP-CpGs), comprising 1800 (1593 + 207) sites uniquely hypermethylated in CIMP-CCs, and (ii) CC-neutral-CpGs (CCN-CpGs), comprising all remaining CpGs (1312; 131 + 1181) showing CC-specific hypermethylation but no CC-subtype specificity (Fig. 1e). Unlike a previous analysis of CIMP cancer methylation [20], where differential methylation was determined by comparing CIMP- to nonCIMP-cancers, the assessment here is based on a comparison of both cancer subtypes to HNM as baseline. This method yielded an additional 571 CIMP-CpGs as well as 1116 previously unidentified CCN-CpGs, showing hypermethylation in all CCs (Fig. 1f). We verified the discrimination power of the newly defined CIMP- and CCN-CpG hypermethylation sites by performing HM27K in an independent set of eight cancers with paired normal mucosa (Fig. 1g)*.* The resulting CIMP- and CCN-CpG-based DNA methylation profiles clustered BRAF^V600E^ cancers separately from BRAF^WT^ cancers and paired normal mucosa, demonstrating the discrimination power of these DNA methylation signatures.

Next, we compared the CC subtype-specific DNA methylation signatures with methylation data available for TAs and SSA/Ps [21, 22]. CIMP-CpGs showed no hypermethylation (< 5% median increase) in nonCIMP-CCs (per definition) and TAs compared to normal mucosa (Fig. 1h) but did show significant hypermethylation (17% median increase, *P =* 7e-239) in SSA/Ps, which was further increased in CIMP-CCs (28% increase, *P* < 2e-16). By contrast, CCN-CpGs showed 20–30% median methylation increase irrespective of cancer- and precursor-subtype when compared to normal mucosa with levels increasing from TAs to nonCIMP-CCs and from SSA/P to CIMP-CCs. Thus, CIMP-CpG and CCN-CpG hypermethylation starts early in CC carcinogenesis with CIMP-CpG methylation discriminating the SSA/P-CIMP from the TA-nonCIMP cancer pathways.

We then asked whether and how age and lifestyle factors affect cancer subtype-specific DNA methylation drift in the HNM. CCN-CpGs, but not CIMP-CpGs, were enriched in sites previously identified as showing age-dependent hypermethylation in the HNM [19] (odds ratio [OR] *=* 6.7, *P* = 2e-129; Fig. 1i). In addition, the median rate of age-dependent methylation gain was higher at CCN-CpGs than at CIMP-CpGs (methylation rate ratio [MRR] = 5.4, *P =* 2e-114). Aspirin use and hormonal replacement therapy (HRT) suppressed the rate of methylation change at CCN-CpGs (MRR_aspirin_ = 0.60, MRR_HRT_ = 0.55) significantly more (aspirin, *P* = 4e-46; HRT, *P* = 9e-61) than at CIMP-CpGs (MRR_aspirin_ = 0.49, MRR_HRT_ = 1.8; Fig. 1j). Taken together, these results suggest that hypermethylation of CCN-CpGs is driven by age and modulated by lifestyle, whereas hypermethylation of CIMP-CpGs appears to follow a different pattern. Given the strong association of colon cancer CIMP with *BRAF*^*V600E*^*,* we explored the role *BRAF*^*V600E*^ as a genetic cause of CIMP-CpG hypermethylation and, hence, colon CIMP.

### TET1 and TET2 are downregulated in BRAF^V600E^-mutated colon cancers, precursor lesions, and cell lines

**Fig. 2:**
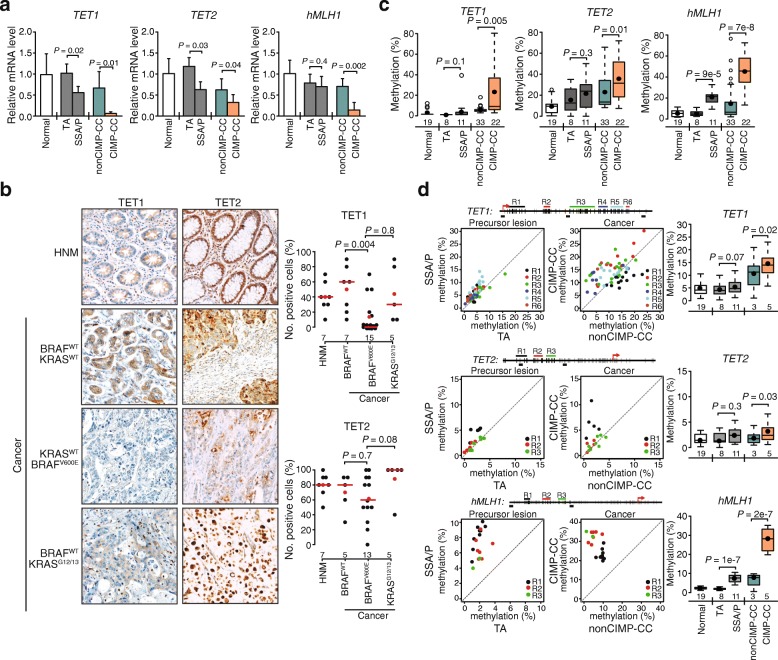
TET1 and TET2 are suppressed in SSA and CIMP-CC carrying BRAF^V600E^. **a***TET1, TET2,* and *hMLH1* mRNA expression, presented as relative expression compared to paired normal mucosa. *P* values were calculated with Welch two sample *t*-test and error bars denote SD. **b** IHC of TET1 and TET2 in healthy normal mucosa (HNM) and cancers with wild type BRAF and KRAS (BRAF^WT^/KRAS^WT^), mutated BRAF (BRAF^V600E^/KRAS^WT^) or KRAS (BRAF^WT^/KRAS^G12/13^). Representative example for each category (*left*) with quantitation (*right*) showing in red mean (circle) and median (line). *P* values were calculated with Wilcoxon rank-sum test. **c** β-values at TET1 and TET2 measured by HM27K. *P* values were calculated with Wilcoxon rank-sum test. **d** DNA methylation at *TET1*, *TET2*, and *hMLH1* promoter-CGIs by bisulfite-pyrosequencing in samples from panel *a*. Schematic shows 6 sequencing regions (R1–R6) within *TET1* (nt − 16 to + 800), 3 sequencing regions (R1–R3) within *TET2* (nt − 140 to + 566) and 3 sequencing regions (R1–R3) with in distal promoter of *hMLH1* (nt − 1080 to + 200) with primer positions (arrows), CpGs (black vertical lines), CpGs not analyzed (grey vertical lines), transcription start sites (TSS, red). Scatter plots of mean methylation levels for each CpG (*left*) and boxplots of the resultant methylation levels (*right*). *P* values were calculated with Wilcoxon rank-sum test. *TA* tubular adenoma, *SSA/P* sessile serrated adenoma/polyp

Molecular mechanisms underlying CIMP in cancer have been intensely investigated. TET as DNA demethylating proteins have emerged as key players in DNA hypermethylation in acute myeloid leukemia, gliomas, and paragangliomas [10–12]. Epidermal growth factor receptor (EGFR) and MAPK activation-mediated silencing of *TET1* was observed in cellular and animal models of lung cancer [23], but the validity of such a mechanism in human lung cancers is uncertain [24]. We investigated the possibility of TET gene dysregulation in BRAF^V600E^-mutated CIMP-CC and found that *TET1* and *TET2* mRNA levels were significantly reduced in SSA/Ps relative to TAs as well as in CIMP-CCs relative to nonCIMP-CCs (Fig. 2a). We also included *hMLH1,* a marker of colon CIMP, in the analysis; *hMLH1* expression was significantly reduced in CIMP cancers but not in SSA/Ps, consistent with its late inactivation in CIMP-CC development. To substantiate TET gene downregulation in CIMP-CCs, we performed immunohistochemical (IHC) analyses; BRAF^V600E^-mutated (by inference CIMP) CCs showed a significantly lower proportion of TET1 expressing cells (median 0%) than KRAS^G12/13^-mutated (median 30%), or BRAF and KRAS wild-type cancers (BRAF^WT^/KRAS^WT^; median 60%; Fig. 2b). The trend was the same for TET2; TET2 positive cells were fewer in cancer with BRAF^V600E^ (median 60%) than without BRAF^V600E^ (KRAS^G12/13^, median 100%; BRAF^WT^/KRAS^WT^, median 80%). Downregulation of *TET1* but not *TET2* in BRAF^V600E^ tumors was confirmed using TCGA RNA-seq data of 274 colon cancers samples from females (Additional file 1: Figure S1). This association seems to be specific for females and not observed when samples from males and rectum were also included.

Notably, *TET1* and *TET2* were hypermethylated in CIMP-CC compared to nonCIMP-CC or normal mucosa but not in precursor lesions, where expression was downregulated (Fig. 2c). As expected, the distal promoter region of *hMLH1*, which acquires methylation early in CIMP carcinogenesis without affecting gene expression [25, 26] was hypermethylated in both SSA/P and CIMP-CC compared to TA and nonCIMP-CC, respectively. We confirmed these findings by bisulfite-pyrosequencing of promoter-associated CGIs (Fig. 2d). These results suggested that *TET1* and *TET2* repression occurs at an early stage in CIMP-CC development, preceding the hypermethylation of their promoters, while *hMLH1* is still expressed. In CIMP-CCs, however, the TET genes gain methylation and are further decreased in expression, suggesting that TET downregulation undergoes epigenetically stabilization during tumor progression.

**Fig. 3:**
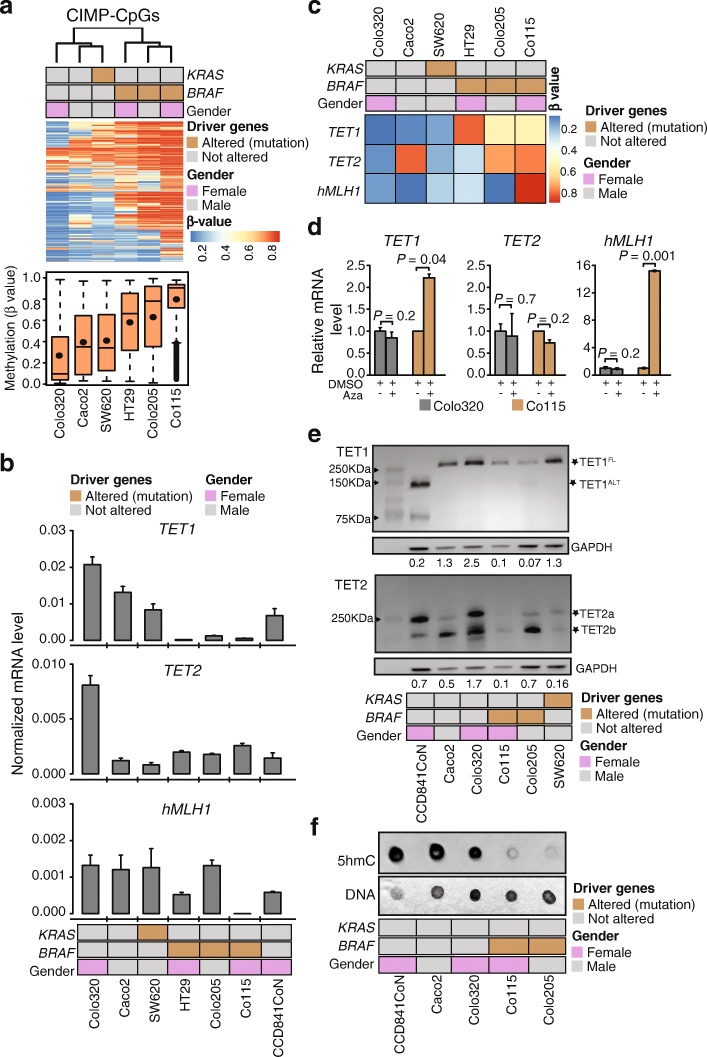
TET1 is suppressed in BRAF^V600E^ cancer cell lines. **a** Unsupervised hierarchical clustering using DNA methylation levels of CCN-CpGs (1312) and CIMP-CpGs (1800) in colon cancer cell lines measured by HM27K/HM450K. Shown are the CpGs that were present on both arrays. Heatmap of the β-values (*upper*) with boxplot of resultant β-values (*lower*). Note the increase DNA methylation in cell lines with BRAF^V600E^. **b***TET1, TET2,* and *hMLH1* mRNA expression in colon cancer and in normal colon epithelial cell lines (CCD841CoN). Error bars denote SD (*n* = 3). Primers were designed to measure both isoforms of TETs. **c** β-values of TET1 and TET2 CpGs measured by HM27K/HM450K. **d***TET1, TET2,* and *hMLH1* mRNA expression following treatment with 0.1 μM 5-azacytidine (Aza) or dimethyl sulfoxide (DMSO), presented as relative expression compared to DMSO. *P* values were calculated with Welch two sample *t*-test. Error bars denote SD (*n* = 3). **e** Western blot analysis of TET1 and TET2. Indicated with asterisks are the full-length (TET1^FL^) and alternative (TET1^ALT^) TET1 and two isoform of TET2 (a, b). Signal was quantified by image studio software and shown as ratio to GAPDH for TET1 (TET1^FL+ALT^) for TET2 (TET2a and 2b). Shown are the representative blot from four (for TET1) or two (for TET2) independent experiments. **f** Levels of 5hmC measured by dot blot analysis with methylene blue staining (DNA) as loading control

We then corroborated the relationship between CIMP, BRAF^*V600E*^ and TET downregulation in CC cell lines. Unsupervised hierarchical clustering on the basis of CIMP-CpGs (Fig. 1e) methylation separated BRAF^V600E^ cell lines (HT29, Colo205, Co115) from BRAF^WT^ (Colo320, Caco2) or KRAS^G12V^ (SW620) cell lines (Fig. 3a). Notably, CIMP-CpGs showed markedly higher methylation in BRAF^V600E^ than in BRAF^WT^ cell lines, while CCN-CpGs were similarly hypermethylated in all cell lines. *TET1* mRNA expression was significantly reduced in all BRAF^V600E^ compared to BRAF^WT^ or KRAS^G12V^ cancer cells or normal colon epithelial cells (CCD841CoN, Fig. 3b), and this downregulation was correlated with increased DNA methylation in the *TET1* promoter (Fig. 3c). *TET2* expression was generally low in all cell lines except Colo320 (Fig. 3b), the *TET2* promoter showed hypermethylation both in BRAF^V600E^ and BRAF^WT^ cell lines (Fig. 3c). As expected, *hMLH1* was downregulated and hypermethylated in the distal promoter in Co115, to a lesser extent in HT29 but not in Colo205 (Fig. 3b, c), consistent with the previously shown heterogeneity of *hMLH1* silencing in *BRAF*^*V600E*^ CIMP cancers and SSA/Ps [13, 27, 28]. Treatment of Colo320 and Co115 cells with the DNA methyltransferases inhibitor 5-azacytidine increased the expression of *TET1* and, as expected, *hMLH1* but did not affect *TET2* (Fig. 3d), demonstrating that DNA methylation directly controls *TET1* rather than *TET2* silencing. Immunoblots confirmed reduced levels of TET1 in Co115 and Colo205 compared to Caco2 and Colo320 (Fig. 3e). Notably, the normal epithelial cell line CCD841CoN showed low expression of full-length TET1 (TET1^FL^) but high levels of an alternative isoform (TET1^ALT^) [29] instead. Immunoblots for TET2 detected both known isoforms with levels varying between nonCIMP and CIMP cell lines (Fig. 3e), as predicted from the variable mRNA expression.

Consistent with reduced TET activity, global levels of 5hmC were lower in the BRAF^V600E^ than in BRAF wild-type cancer cells or normal colon epithelial cells (Fig. 3f). These results show that CC cell lines partially recapitulate the TET expression and promoter methylation features of cancers with a corresponding BRAF mutation status, in particular the consistent repression of TET1^FL^ in the presence of a BRAF^V600E^.

### BRAF^V600E^ represses TET and causes hypermethylation at CIMP genes

**Fig. 4:**
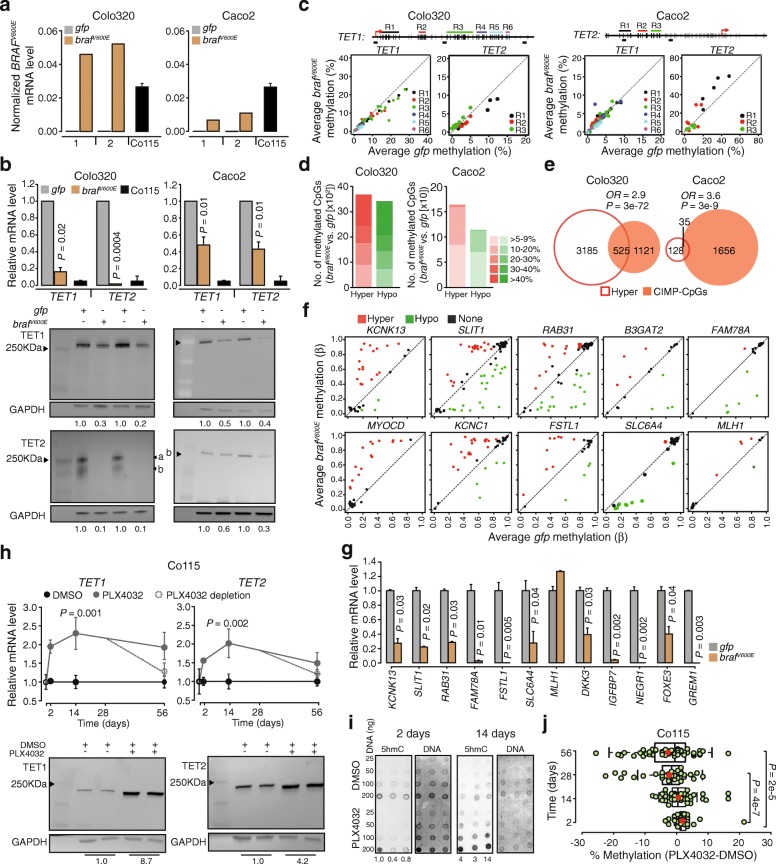
Ectopic expression of BRAF^V600E^ represses TET1 and TET2 and causes DNA hypermethylation. **a** Lentiviral BRAF^V600E^ mRNA expression in BRAF^V600E^ (*braf*^*V600E*^) and control (*gfp*)-transduced Colo320 and Caco2 cell lines normalized to *GAPDH* and *ACTB*. Co115 cell constitutively expressing BRAF^V600E^ is used as reference. **b***TET1* and *TET2* mRNA expression (*upper*) with Western blot analysis of protein levels (*lower*) in cells from panel *a*. *P* values were calculated with Welch two-sample *t*-test. Error bars denote SD (*n* = 2). Protein signal quantified by image studio software is relative to *gfp*. Shown are the representative blot from two independent experiments. **c** DNA methylation at *TET1* and *TET2* promoter-associated CGIs by bisulfite-pyrosequencing in cells from panel *a*. Representation is as in Fig. 2b. **d** Genome-wide methylation profiles in cells from panel *a*. Shown are the number of hyper (red) and hypo (green) methylated CpGs. To make analysis comparable between platforms, only CpGs corresponding to HM27K are shown. **e** Venn diagrams show overlap of hypermethylated CpGs from panel *d* with CIMP-CpGs identified in colon cancers in Fig. 1. Calculated Fisher's exact test are reported as well as associated odds ratios. **f** Methylation levels at CIMP markers of a panel previously proposed by Hinoue and coworkers (*B3GAT2*, *KCNK13*, *RAB31*, *SLIT1*, *FAM78A*, *FSTL1*, *KCNC1*, *MYOCD*, and *SLC6A4*) and *hMLH1* in Colo320 *braf*^*V600E*^ and *gfp* cells from panel *a*. Shown are all CpGs present on the array for the corresponding gene; hypermethylated (red), hypomethylated (green) or none (black). **g** The mRNA expression levels of 12 CIMP-CpG-associated genes in *braf*^*V600E*^ cells relative to *gfp. P* values were calculated with Welch two sample *t*-test. Error bars denote SD (*n* = 2). **h***TET1* and *TET2* mRNA expression (upper) in Co115 cells treated with 2 μM PLX4032 (grey) or DMSO (black) for 56 days. At 28 days, cultures were continued with (straight line) or without PLX4032 (dotted line). Shown are expression levels relative to DMSO. Day 0 is shown in open circles. *P* values were calculated with Welch two sample *t*-test. Error bars denote SD (*n* = 5). Western blot for TET1 and TET2 at 14 days in two represented replicates. Protein signal quantified by image studio software is relative to DMSO. Shown are the representative blot from three independent experiments. **i** Dot blot showing levels of 5hmC at 2 and 14-day timepoints in cells from panel *h*. Shown are 3 replicates for each timepoint with methylene blue staining (DNA) as loading control. Signal quantified by image studio software is a shown as ratio (PLX4032/DMSO). **j** DNA demethylation at the *TET1* promoter-CGI in cells from panel *h*. Boxplots show the methylation difference (PLX4032-DMSO) measured by bisulfite-pyrosequencing at 61 CpGs (green circles) with median (line) and mean (red circles). *P* values were calculated with Wilcoxon rank-sum test

To investigate whether BRAF^V600E^ is sufficient for *TET1/TET2* repression and hypermethylation at CIMP-CpGs, we transduced Colo320 and Caco2 cells with a lentivirus expressing BRAF^V600E^ (*braf*^*V600E*^) or a GFP (*gfp*) as a control. Both these CC cell lines are wild types for BRAF and showed low levels of CIMP-CpG methylation (Fig. 3a). Expression of *BRAF*^*V600E*^ was confirmed at day 14 following transduction; relative *BRAF*^*V600E*^ expression reached higher levels in Colo320-*braf*^*V600E*^ than in Caco2-*braf*^*V600E*^ but was in a plus/minus two-fold range of levels observed in Co115 with constitutive BRAF^V600E^ expression (Fig. 4a). BRAF^V600E^ caused downregulation of *TET1* and *TET2* in both cell lines with magnitude of downregulation inversely correlating with *BRAF*^*V600E*^ expression (Fig. 4b). Bisulfite-DNA sequencing revealed that CGIs in the *TET1* and *TET2* promoters, showing hypermethylation in SSA/P and CIMP-CC (Fig. 2d), did not gain methylation upon ectopic expression of BRAF^V600E^ (Fig. 4c).

We then addressed the effect of *BRAF*^*V600E*^ expression on genome-wide DNA methylation. *BRAF*^*V600E*^-transduced cell lines, when compared to their respective *gfp* controls, exhibited widespread gains (hyper) and losses (hypo) of DNA methylation (Fig. 4d). CpGs undergoing hypermethylation in both BRAF^V600E^-transduced cell lines showed a significant overlap with CIMP-CpGs identified in CCs (Fig. 4e). Included in this overlap were 9 out of 10 CIMP markers of a panel previously proposed by Hinoue and coworkers [20] (*B3GAT2*, *KCNK13*, *RAB31*, *SLIT1*, *FAM78A*, *FSTL1*, *KCNC1*, *MYOCD*, and *SLC6A4*). When assessed methylation change at single CpG resolution at the promoters of these genes, discontinuous patterns of methylation, including both hyper- and hypomethylation were observed (Fig. 4f) that altogether were associated with downregulated expression of the respective genes in *braf*^*V600E*^ vs. *gfp*-control (Fig. 4f). Expression analysis of five additional CIMP-CpG-associated genes identified in Fig. 1 (*DKK3*, *IGFBP7*, *NEGR1*, *FOXE3*, and *GREM1*) also showed downregulation in *braf*^*V600E*^-transduced cells (Fig. 4g). *hMLH1*, showing some hypermethylated CpGs on its distal promoter in *braf*^*V600E*^-transduced cells, was not downregulated (Fig. 4g). Consistently, MAFG, a transcriptional repressor that was shown to mediate silencing of *hMLH1* in CC, was not induced in the *braf*^*V600E*^-transduced cell lines (Additional file 1: Figure S2).

Next, we tested whether inhibition of BRAF^V600E^ would restore *TET* expression. Treating Co115 cells with a sub-toxic concentration of PLX4032 (Vemurafenib, 2 μM), a specific BRAF^V600E^ inhibitor, increased *TET1* and *TET2* mRNA levels after 2 days and up to 56 days of treatment (Fig. 4h), as well as protein levels measured at 14 days of treatment (Fig. 4h). The effect of PLX4032 was reversible; withdrawal of the drug after 28 days was accompanied by a reduction of *TET* transcripts to starting levels (Fig. 4h). PLX4032 treatment resulted in no detectable change in global 5hmC at 2 days but showed a pronounced increase at 14 days (Fig. 4i). Notably, PLX4032 treatment also decreased *TET1* promoter methylation in a time-dependent manner (Fig. 4j). Taken together, these results show that ectopic BRAF^V600E^ expression transcriptionally downregulates *TET1* and *TET2* independent of methylation changes in their promoter. TET inactivation then gives rise to DNA methylation changes that include the hypermethylation and silencing of typical CIMP target genes. Hypermethylation at TET promoters appears to be a progressive and later event that stabilizes their silenced state in CIMP tumorigenesis.

### Oncogenic BRAF expression in the mouse small intestine causes TET silencing and DNA hypermethylation

**Fig. 5:**
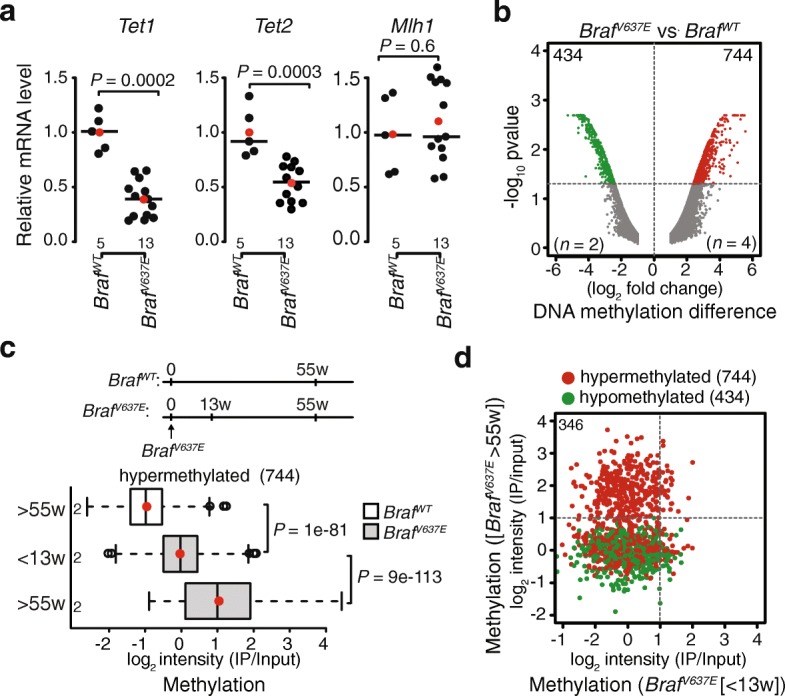
TET1 and TET2 are repressed in conditional *Braf*^*-V637E*^ knock-in mice. **a***TET1, TET2,* and *Mlh1* mRNA expression in the proximal small intestine of mutant *Braf*^*V637E*^ mice (*n* = 13) relative to wild-type *Braf*^*WT*^ mice (*n* = 5). Shown are the median (line) and mean (red circle). *P* values were calculated with Welch two sample *t*-test. **b** Differential methylated probes either hypermethylated (red) or hypomethylated (green) in *Braf*^*V637E*^ mice versus *Braf*^*WT*^ mice. Numbers of probes are at the top and number of samples are shown as *n*. **c** Experimental set up underlying samples used for genome-wide methylation analysis on mouse tilling array (NimbleGen) taken from mice either at less than 13-week (< 13 weeks) or more than 55-week (> 55 weeks) timepoint (*upper*). Box plots showing average DNA methylation at hypermethylated probes (744) in *Braf*^*WT*^ and *Braf*^*V637E*^ mice at < 13- or > 55-week timepoints. Plotted are the input-normalized intensity levels on *y*-axis (log_2_) with medians (line) and means (red circle). *P* values were calculated with Wilcoxon rank-sum test. **d** Scatter plot showing methylation in *Braf*^*V637E*^ mice at < 13 or > 55-week timepoint. Plotted are the input-normalized intensity levels (log_2_) of hypomethylated (green) or hypermethylated (red) DMPs. The 346 probes methylated at > 55-week timepoint are indicated in the box. *P* values were calculated with Wilcoxon rank-sum test

To recapitulate BRAF-dependent TET silencing *in vivo*, we examined tissues from a previously established murine *Braf*^*LSL-V637E/+*^*Vil-Cre*^*+/-*^ knock-in mouse model [30]. The V637E mutation in mouse Braf is functionally equivalent to the V600E mutation in human BRAF, and the *Vil-Cre* transgene facilitates the Cre-induced activation of *Braf*^*LSL-V637E*^ specifically in the epithelia of the small and large intestine of the knock-in mice [31]. *Braf*^*V637E*^ expression in these mice gave rise to extensive, generalized, and persistent hyperplasia in the intestine [31]. We examined Tet1 and Tet2 expression in the mucosa of the proximal small intestine from mutant *Braf*^*V637E*^ (mean age 60 weeks) and *Braf*^*WT*^ mice (mean age 64 weeks). *Tet1* and *Tet2* mRNA levels were significantly lower in the hyperplastic *Braf*^*V637E*^ mucosa when compared to the normal mucosa of wild-type mice; *Mlh1* expression was not affected (Fig. 5a). As in human SSA/Ps, transcriptional repression of the *Tet* genes was independent of hypermethylation of their promoter CGIs (Additional file 1: Figure S3). Yet, *Tet* repression was accompanied by changes in DNA methylation elsewhere. Analysing six tissue samples on mouse CGI plus promoter tiling arrays (Roche NimbleGen Inc.), we identified 1178 probes showing differential methylation between *Braf*^*WT*^ and the *Braf*^*V637E*^ mice. Amongst these, 744 were hypermethylated and 434 hypomethylated in the *Braf*^*V637E*^ mice (Fig. 5b). Notably, the median methylation level was significantly higher in *Braf*^*V637E*^ older mice (> 55 weeks) than in younger mice (< 13 weeks), consistent with a recent observation of a gradual increase in DNA methylation following *Braf*^*V637E*^ induction in mice [32]. Three-hundred-forty-six probes showed hypermethylation only in tissue of > 55-week-old mice, and the methylation at these sites occurred only in *Braf*^*V637E*^ but not *Braf*^*WT*^ mice of the same age, suggesting that the underlying cause is the *Braf* mutation. Taken together, these results demonstrate that persistent oncogenic *Braf* signalling is sufficient to deregulate TET expression and induce progressive widespread DNA methylation changes.

### BRAF^V600E^-TET directed targeted DNA hypermethylation has the potential to drive CIMP carcinogenesis

**Fig. 6:**
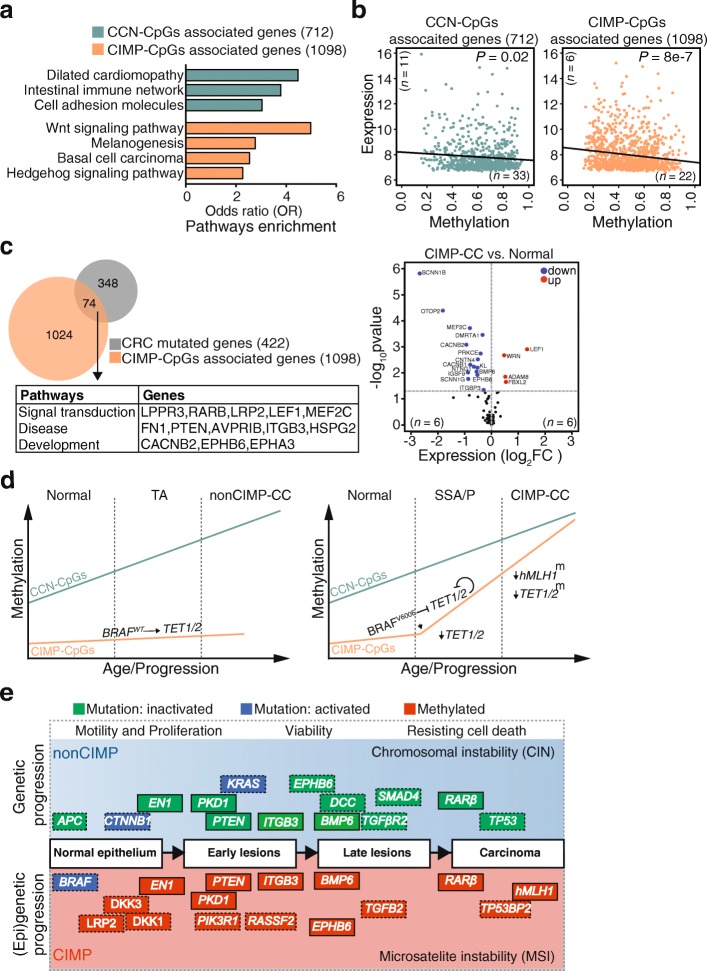
Functional correlation of CC-subtype specific methylation. **a** Enriched KEGG pathways (*P* < 0.01) within CCN-CpGs and CIMP-CpG-associated genes. Shown are only those pathways that were enriched exclusively. **b** Correlation between methylation and expression at CCN-CpGs and CIMP-CpG-associated genes in nonCIMP-CC or CIMP-CCs. Number of samples are shown as *n*. *P* values are calculated as Pearson correlation*.***c** Overlap of CIMP-CpG-associated genes with genes acquiring mutations during colon tumorigenesis (*left*) and differential gene expression of these overlapped genes (74) in CIMP-CCs versus paired normal mucosa. Shown are log_2_ fold changes (*x*-axis) versus −log_10_ false discovery rate (FDR)-adjusted *P* value (*y*-axis). Colored dots represent significant (FDR-adjusted *P* < 0.05) upregulated (red) or downregulated (blue) genes and number of samples are shown as *n*. **d** Model depicting changes in methylation at CCN-CpGs and CIMP-CpGs from healthy normal mucosa through precursor lesions to cancer as described in text. *TA* Tubular adenoma, *SSA/P* sessile serrated adenoma/polyp. (), downregulated gene expression; ^m^, methylated; (), inhibition; (), feedback inhibition. **e** Epigenetic progression model for subtype-specific colon carcinogenesis. Illustration depicting identical (solid rectangle) or functionally equivalent (dotted rectangle) genes affected by genetic mutation in nonCIMP (blue) or by epigenetic deregulation in CIMP (red) carcinogenesis. Normal epithelium to carcinoma progression is depicted by rectangles with arrowheads. Genomic instability (CIN or MSI) associated with specific tumor subtypes is indicated

Pathway analyses of CIMP-CpG-associated genes revealed a specific functional link with developmental pathways often mutated in colon cancer [33–36], such as WNT (wingless-related integration site), HH (hedgehog), and basal cell carcinoma (TGF and p53 signalling pathways). By contrast, CCN-CpG-associated genes were related to genes of the intestinal immune network, cell adhesion, and cardiomyopathy function (Fig. 6a). Hypermethylation at CIMP-CpGs, much less at CCN-CpG, correlated inversely with mRNA expression at associated genes (Fig. 6b), corroborating a functional impact of CIMP-CpG hypermethylation on these genes and, hence, pathways. These observations resemble the molecular and functional features associated with two main consensus molecular subtypes (CMS1/CMS2) of colon carcinogenesis, recently identified on the basis of gene expression analyses [16, 17]. Intersecting genes previously identified as acquiring mutations in colon tumorigenesis [37] with CIMP-CpG-associated genes, identified 74 genes with functions in signal transduction (*LEF1*, *MEF2C*, *RARB*), disease (*PTEN*, *ITGB3*, *FN1*), and development (*EPHB6*, *EPHA3*). Notably, many of these genes, including the tumor suppressors *BMP6, EPHB6, ITGBP3,* were downregulated in CIMP-CC (Fig. 6c). Together, these data suggest that epigenetic dysregulation can compensate for genetic mutation to drive CIMP cancer progression.

## Discussion

Carcinogenesis follows evolutionary principles whereby progressive genetic and epigenetic change creates patterns of molecular dysregulation that cause heterogeneous subtypes of disease. In CC, the “classical” adenoma-carcinoma sequence is well aligned with progressive genetic mutation [2], but the contribution of epigenetic change, most prominent in CIMP-CC developing through the “serrated” pathway, has remained elusive. In this study, we compared CC-subtypes with HNM to define pan-CC-specific DNA methylation changes. This approach, in particular, identified two classes of CpGs with distinct hypermethylation properties in tumorigenesis along the “classical” and the “serrated” pathways caused by distinct underlying mechanisms. CCN-CpGs showed hypermethylation in all CCs, had comparably high base levels of methylation in the HNM that are subject to change mainly through an age-dependent, lifestyle-modulated process. CIMP-CpGs, on the other hand, were hypermethylated specifically in CIMP-CCs, showed low-base-level methylation in the HNM and gain significant methylation only through genetically controlled repression of TET1 and TET2 DNA demethylases, which is apparent already in SSA/P precursors.

Given that CIMP is highly correlated with female gender [38] and CC has features distinct from rectal cancer [39], we restricted our analysis to the female gender and the proximal and distal colon (no rectum), and this applied also to all published data sets used in this study, including the 56 out of 125 cancer samples from Hinoue and coworkers [20]. This was to increase discriminative power within the cohort. Consistently, as CIMP-low methylation is significantly more common in men [40], our cluster analysis did not reveal this category. Therefore, our observations and conclusions cannot be directly extended to the male population.

While the *BRAF*^*V600E*^ mutation has been correlated with TET silencing [13] and associated with SSA/P specific DNA methylation [41], the underlying causality has not been established experimentally. Here, we establish BRAF^V600E^ as the cause of transcriptional repression of TET DNA demethylases, which generates a reversible hypermethylation phenotype early in CC carcinogenesis. The factor(s) causing BRAF^V600E^ mutation and the initial dysregulation of TET1 and TET2 in response to BRAF^V600E^ activation, still remains to be determined. It was shown recently that BRAF^V600E^ induces silencing of *hMLH1* and other CIMP genes through phosphorylation of the transcriptional repressor MAFG [42]. We therefore investigated whether BRAF^V600E^-induced repression of *TET* genes is MAFG mediated as well. Ectopic BRAF^V600E^ expression in our CC cell models did not alter MAFG levels (Additional file 1: Figure S2) nor did it repress *hMLH1* (Fig. 4g)*,* but it did repress *TET* and CIMP marker genes (Fig. 4b, g). In addition, repression of *TET* genes was apparent in SSA/Ps that still expressed *hMLH1* (Fig. 2a). Consistent with these observations, it has been shown that silencing of *hMLH1* in CIMP carcinogenesis is a relatively late event [6, 43, 44]. We therefore conclude that MAFG is not responsible for *BRAF*^*V600E*^-induced *TET1* and *TET2* repression and CIMP-CpG hypermethylation early in CC tumorigenesis but may become relevant later in CIMP carcinogenesis for silencing of *hMLH1* and other CIMP genes. The identification of *BRAF*^*V600E*^ mutated CIMP cancers lacking *hMLH1* methylation and MSI [27] further documents that CIMP and *hMLH1* silencing can be uncoupled and therefore do not have a single common underlying defect. *TET* silencing may thus contribute to CIMP-mediated tumorigenesis in CC that may or may not include *hMLH1* methylation [13].

Indeed, clinical data suggest that CIMP is established early in the “serrated” CC pathway and is associated with older patient age [27]. We show that tissue methylation levels at CIMP-CpGs increase from BRAF^V600E^ SSA/P to CIMP-CC in humans and from Braf^V637E^ young to old mice. This suggests that mutated BRAF-mediated *TET* repression is a prerequisite for early CIMP establishment, but not per se determining SSA/P progression. Rapid transition to cancer was suggested to occur in dysplastic SSA/P, in conjunction with loss of *hMLH1* expression, following a prolonged dwell time of SSA/P without dysplasia [45]. Our data are consistent with *hMLH1* inactivation occurring late in CIMP-carcinogenesis, subsequent to BRAF^V600E^-mediated TET repression in SSA/P. It is therefore plausible that *hMLH1* hypermethylation and silencing is a late consequence of BRAF^V600E^-induced TET repression in SSA/P, which then define the onset of a mutator phenotype and a rapid progression to cancer. This may explain the over-representation of CIMP and MSI in post-colonoscopy CC (PCCC) [46, 47]. We therefore propose that stable *TET1* and/or *TET2* silencing by promoter methylation is a risk factor for *hMLH1* silencing and PCCC.

Previously, stable transfection of BRAF^V600E^ in Colo320 cells showed no overall increase in DNA hypermethylation [48]. Assessing the methylation status 14 days following *BRAF*^*V600E*^ transduction, however, we observed widespread gains and losses of DNA methylation (Fig. 4d). Amongst hypermethylated CpGs were several genes of the CIMP marker panel defined by Hinoue and coworkers (Fig. 4f) [20]. There are several possible explanations for these discrepancies; (i) previous analysis [48] was done by the GoldenGate array (illumina) technology, which has far lower CpG representation compared to the EPIC array (1536 vs. > 850,000) used for analyses, (ii) genes that acquire methylation later in tumor progression may not show differential methylation in short-time-course experiments performed in cell culture; i.e. timing and culture conditions may have been different in the experiments.

Based on our findings, we propose a model whereby DNA hypermethylation at CCN-CpGs is mainly an effect of tissue aging and exposure that accompanies carcinogenesis through the “classical” pathway. By contrast, hypermethylation at CIMP-CpGs is the result of a genetically controlled, deterministic mechanism that shapes carcinogenesis through the “serrated” pathways (Fig. 6d). Overall, the data suggest a stepwise establishment of CIMP-CC. As the *TET1* and *TET2* promoters are both targets for TET1 binding [49] and TET-dependent demethylation themselves [50, 51] (Additional file 1: Figure S4), initial BRAF^V600E^-induced repression of the *TET* genes will predisposes their promoters to hypermethylation, which will epigenetically stabilize their repressed state. Ultimately, *TET1* and *TET2* silencing causes a widespread DNA demethylation defect at *TET1*/*TET2* targeted loci and, hence, establishes full-blown, stable CIMP detectable in CC (Fig. 6d). Progressive hypermethylation can affect and silence the promoter of *hMLH1* at a later stage, thereby aggravating genetic instability by establishing a mutator phenotype. Exactly how differential hypermethylation contributes to subtype-specific CC initiation, progression, and clinical heterogeneity, including the anatomic location and gender predilection of CIMP-CC, remains unclear. Notably, however, the epigenetic dysregulation ensuing by BRAF^V600E^-driven CIMP has the potential to effect functional changes along the “serrated” CC pathway that are achieved by genetic mutation in the “classical” pathway (Fig. 6e). Differential contributions of aberrant DNA methylation and genetic mutation establish functionally equivalent changes in key pathways of carcinogenesis and, yet, shape characteristic phenotypes of CC subtypes.

## Conclusions

Our data indicate an intimate functional crosstalk between genetic mutation and epigenetic aberrations, particularly in the “serrated” pathway. This work is built upon and expands existing knowledge about the CIMP and act as conceptual framework that will help unravel the functional significance of CIMP in colon cancer and elsewhere. Besides this conceptual advance, the finding that age- and genetically-driven DNA hypermethylation shows distinct kinetics, contributions, and patterns in nonCIMP- and CIMP-CC has clinical implications. The identification of CC subtype-specific DNA methylation signatures has clinical relevance for identifying biomarkers in the assessment of subgroup-specific cancer risk and disease progression and to improve preventive and early detection interventions in CC. Further exploration of important insights into the mechanisms by which BRAF^V600E^ regulates TET1 serve as a knowledge base that can be exploited for therapeutic benefit.

## Methods

### Establishment of stable BRAF^V600E^ cell lines

Colo320 and Caco2 cells stably expressing oncogenic BRAF were established using full-length BRAF^V600E^ cloned into self-inactivating bicistronic lentivirus expression vector (PLV401) containing the CMV promoter via LR reaction (Invitrogen). The plasmid with eGFP only was used as control. Both plasmids were kindly provided by Dr. G. Lizee, Department of Melanoma Medical Oncology, University of Texas, and details are described previously [52]. Expression vectors were co-transfected with pCMV-VSV-G (Addgene, 8454) and pCMV-dR8.2 dvpr (Addgene, 8455) into HEK293T cells using Lipofectamine 2000 (Invitrogen). Viral supernatants were collected at 48, 72, and 96 h, pooled and concentrated using the Lenti-X Concentrator (Clontech) according to manufacturer instructions. Lentiviral particles were quantified by means of the Lenti-X p24 rapid titre ELISA Kit (Clontech). Aliquots of viral particles were frozen at – 80 °C. For lentivirus transduction, Colo320 in RPMI-1640 and Caco2 in Eagle's minimum essential medium (EMEM) were cultured in a 24-well plate at a density of 1 × 10^5^ cells/well 24 h before transduction. Cells were incubated with lentivirus-containing medium supplemented with 8 μg/ml polybrene (Sigma-Aldrich) for 24 h. After exchanging with fresh medium, cells were grown for 14 days. All further experiments were carried out 14 days after transduction in two independent cell populations either stably expressing BRAF^V600E^ (*braf*^*V600E*^) or eGFP control (*gfp*).

### Western blot

Cells were lysed for 30 min on ice in lysing buffer (50 mM Na-P buffer pH 8, 125 mM NaCl, 1% NP-40, 500 μM EDTA, 1 mM DTT, 1 mM PMSF) supplemented with 1 cOmplete EDTA-free protease inhibitor cocktail solution (Roche) and 1X phosphatase inhibitor (PhosStop, Roche). Supernatant was collected after lysate centrifugation at 4 °C for 30 min at 12,000 rpm. Protein concentrations were determined with Bradford assays (Bio-Rad). Equal amount of protein (40 μg) were loaded into polyacrylamide gels (4–20% Mini-Protean TGX Precast gels, Bio-Rad). Candidate proteins were detected with antibodies against TET1 (Abiocode: R1084-1, Sigma: SAB2700730), TET2 (Abiocode: R1086-2b), and MAFG (Biotechne: MAB3924) proteins. GAPDH (Sigma: G9545) serves as loading control. Total 2–4 independent blots were performed with each antibody per experiment condition.

### qRT-qPCR

Total RNA was extracted using RNeasy Mini Kit (Qiagen), and reverse transcription was performed by RevertAid First Strand cDNA Synthesis system (ThermoScientific), followed by qRT-PCR using QuantiTect SYBR Green Kit (Qiagen). *ACTB* and *GAPDH* were used as internal references for normalization. Primers were not isoform specific and therefore measure expression of TET1 (TET^FL^ and TET1^ALT^) and TET2 (isoform a,b) collectively. See Additional file 1: Table S1 for primer sequences.

### Cell cultures and drug treatments

The colon cancer cell lines were grown in growth medium according to ATCG supplemented with 20% fetal calf serum (FCS, Sigma), 1% Penicillin/streptomycin (P/S, Sigma) and 200 mM L-GlutaMax (Sigma). For drug treatment, Co115 cells (5 × 10^6^ cells/15-cm dish) were cultured in growth medium containing 5% serum with 2 μM of PLX4032 (Selleck chemical) or DMSO (vehicle) for 56 days with fresh media changes with drug or vehicle every day. After 28 days, cells were cultured with or without further addition of drug and vehicle until 56 days. Co115 and Colo320 cells were treated with 0.1 μM of 5-Aza-cytidine (Aza) or DMSO (vehicle) for 5 days with fresh media changes with drug or vehicle every day.

### Dot blot assay

Dot blots were performed using antibodies of 5-hydroxymethylcytosine (5hmC; Active Motif: 39769). Briefly, genomic DNAs were blotted onto a H-bond N+ nylon membrane (Amsherham) and dried for 15 min. Membrane-bound DNA was denatured in 400 mM NaOH for 4 h. Membrane was washed twice with SSC buffer pH 7 (300 mM NaCl, 34 mM sodium citrate) and blocked with 10% milk in TBST (20 mM Tris-HCl pH7.5, 150 mM NaCl, 0.1% Tween 20) for 1 h at room temperature (RT). After incubation, membrane was washed three times with TBST. 5hmC was detected with antibodies anti-5hmC antibody (5hmC; Active Motif: 39769). To ensure equal spotting of total DNA on the membrane, the same blot was stained with 0.02% methylene blue in 0.3 M sodium acetate (pH 5.2).

### Bisulfite pyrosequencing

Bisulfite-converted DNA was used to measure methylation levels by pyrosequencing as described previously [19]. See Additional file 1: Table S2 for primer sequences.

### Genome-wide DNA methylation analysis in human samples and cell lines

#### Primary cancers, paired normal mucosa, and cancer cell lines data

Genome-wide DNA methylation of primary cancers (*n* = 8) and 7 cm proximal to the primary cancer adjacent normal mucosa (paired normal, *n* = 8) were measured using HM27K array. Samples were obtained from colon cancer patients undergoing surgical resection at the department of surgery, canton of Aargau, Switzerland under the ethical approval (Ref.Nr. EK: 2004/053). All patients gave their informed consent for the use of their specimens for research purposes. All samples were stored at – 80 °C in RNAlater until further processing. All primary cancers were histologically confirmed adenocarcinomas.

Statistical analyses were performed on logit transformation of β-values known as M-values [53], whereas β-values were used for biologic interpretation. For probe-wise differential methylation analysis, a model adjusting for colonic location and batch effect was fitted, using the limma package [54]. Statistical tests are performed as described in figure legends, and when possible, adjusted *P* values calculated by limma were used to assess for significance, with a threshold of adjusted *P* < 0.05.

Methylation profiles for Caco2, Co115, Sw620 were generated using HM450K array. Illumina GenomeStudio software was used to extract the raw signal intensities of each CpG. All computational and statistical analyses were performed using R and Bioconductor. All preprocessing, correction and normalization steps were performed using complete pipeline adapted from methylumi and lumi R packages as described earlier [55]. Background correction was performed based on un-hybridized negative control probe intensities, and then, background-subtracted signal intensities were normalized with DASEN [55]. To make results comparable between different arrays, CpGs corresponding to HM27K array were used further. Methylation levels at CCN-CpGs and CIMP-CpGs were used for the analysis.

#### Stable-cell lines COLO320 and Caco2 data analysis

Genomic DNA was extracted using QIAamp DNA mini kit (Qiagen) according to the manufacturer's instructions. DNA bisulfite conversion was carried out using EZ DNA Methylation kit (Zymo Research) by following manufacturer's manual. Bisulfite-converted DNA was analyzed using Illumina's EPIC array (for Colo320) and HM450 (for Caco2). Illumina GenomeStudio software was used to extract the raw signal intensities. R and the Bioconductor packages minfi (for EPIC) and methylumi- and lumi-based complete pipeline (for HM450) were used to process and normalize the raw data. Probes with poor signals (*P* > 0.01) were not included. All probes were matched to the human GRCh37/hg19. Chromosome X- and Y-linked probes were removed from subsequent analysis.

For Colo320-transduced cell populations, any CpG was called differentially methylated CpGs if it was significantly (FDR adjusted *P* < 0.05) differentially methylated in *braf*^*V600E*^ versus *gfp* with mean β-value methylation difference > 10%. Since in Caco2, transduction efficiency was low, leading to more heterogeneous cell population, mean β-value of both replicates resulted in no significant differential methylated CpG in *braf*^*V600E*^ versus *gfp*. To account for the variability between replicates, we then performed pair-wise analyses in which each experimental sample was compared to its respective GFP control. A CpG was called differential methylated, if the β-value methylation difference between *braf*^*V600E*^ and respective control gfp was > 5% in both replicates. In order to compare EPIC and HM450K with HM27K, we only selected those probes that were measured on all three platforms.

#### Public healthy colon, primary cancers, and lesions data analysis

Genome-wide DNA methylation of healthy normal mucosa samples (HNM, *n* = 178), tubular adenomas (TAs, *n* = 14) with 6 paired normal, and primary colon cancers (*n* = 56) were obtained from Gene expression Omnibus (GEO; GSE48988 [19], GSE48684 [22], GSE25062 [20]). Methylation was profiled by Illumina HM27K or by HM450K array. DNA methylation of additional TAs (*n* = 8) and sessile serrated adenomas/polyps (SSA/Ps, *n* = 11) with paired normal mucosa were profiled by bisulfite sequencing (E-MTAB-6952) [56].

Clustering analysis was performed by recursively partitioned mixture model (RPMM) on most variable CpG sites (5254) across the cohort, with variability ranked by standard deviation (SD > 0.16). This algorithm was implemented using the RPMM Bioconductor package. For probe-wise differential methylation analysis, a model adjusting for colonic location and batch effect was fitted using the limma package [54]. Statistical analyses were performed on logit transformation of β-values known as M-values [53], whereas β-values were used for biologic interpretation. *P* values were adjusted to control for the false discovery rate (FDR) using the Benjamini–Hochberg method. For the log_2_ fold change (logFC) calculation, the differences between the averages of groups were considered. Significantly differentially methylated CpGs in cancer subgroups were defined as those having an adjusted *P* < 0.0001, logFC > 2 and absolute methylation difference to healthy colon samples > 10%. The CpGs differentially methylated in both cancer subgroups (common) were further tested as following: if common DMC was significantly (*P* < 0.01) more methylated (absolute methylation difference > 10%) in CIMP than nonCIMP cancers, it was then defined as CIMP-CpG otherwise CCN-CpG.

Methylation in precursor lesions was measured either by HM450K (TAs; GSE48684 [22]) or by bisulfite sequencing (TAs and SSA/Ps; E-MTAB-6952 [56]). To account for different detection limit between two platforms, we used absolute methylation difference between precursor lesions to the paired normal mucosa profiled on the same platform. For bisulfite sequencing data, methylation levels corresponding to CCN-CpGs and CIMP-CpGs were computed. UCSC lift over function was used to convert the hg18 CpG sites coordinates to hg19. Methylation proportions (range 0 to 1) were determined as counting number of methylated reads/ total number of reads. The median methylation levels between lesions and cancers were compared by Wilcoxon test. Age- and lifestyle-associated hypermethylated CpGs were identified as described previously. Methylation rate ratio (MRR) was calculated as the rate of CCN-CpGs/rate of CIMP-CpGs or rate of users/rate of nonusers. Methylation profiles of Colo320, HT29, and Colo2015 were published (GSE35573) [57]. Methylation levels at CCN-CpGs and CIMP-CpGs were used for the analysis.

### Genome-wide DNA methylation analysis in conditional BrafLSL-V637 knock-in mice

Mucosa from proximal small intestine was sampled from previously established murine Vil-Cre+/-; *Braf*^*LSL-V637E/+*^ knock-in mice and control *Braf*^*WT*^ mice [31]. Six samples were used to generate genome-wide DNA methylation profiles using Roche NimbleGen Mouse DNA Methylation 3x720K CpG Island plus RefSeq Promoter Arrays. The array can assay 20,404 promoter regions, 22,881 transcripts, and 15,980 CpG Islands in mouse. Experimental-enriched and genomic input fractions for each sample were labelled with Cy5 and Cy3, respectively, following instructions in the NimbleGen Array User Guide DNA Methylation Arrays (Version 7.2). Labelled fractions were pooled and co-hybridized to the arrays. Following hybridization and washing, arrays were scanned using NimbleGen MS 200 Microarray Scanner. For each array feature, a scaled log_2_ ratio was calculated as the ratio of the input signals intensity for the experimental and control samples co-hybridized. Scaling was performed using Tukey-bi-weight scale. Differentially methylated probes between *Braf*^*V637E*^ and *Braf*^*WT*^ were identified by comparing log_2_ intensity ratios for each probe. Probes were analysed individually, rather than aggregated into larger windows or collapsed by gene promoter, in order to retain high resolution of the tiling array platform and to detect region-specific changes that may be masked by analysis of larger, smoothed windows. Probe sequence represented in the mouse genome only once were selected. This resulted 673,940 probes for further analysis. All analyses were performed using R packages Ringo and limma. Array probes were considered differentially methylated at adjusted *P* < 0.05 and a logFC > 2. The mm9 genome build was selected for the analysis.

### Gene expression analysis

Gene expression (GSE25070) [20] of 17 cancers and in paired normal mucosa was measured previously by Illumina Ref-8 whole-genome expression BeadChip. Probe-wise differential expression analysis was performed using the limma package. FDR-adjusted *P* < 0.05 was considered as significant difference.

### TET expression analysis using TCGA data

The Z-scores of mRNA expression data from colon cancer studies were retrieved from the Cancer Genomics Data Server (CGDS) through the cBioPortal for Cancer Genomics http://www.cbioportal.org, using the CGDS-R package. Z-scores were available for 274 female colon cancer samples (BRAF^V600E^; *n* = 46, KRAS^G12/13^; *n* = 94, BRAF^WT^/KRAS^WT^; *n* = 134), whose mRNA expression data were produced on the same platform (RNA-seq , illumin). The scores were calculated using cancer diploid for each gene as the reference population, and individual overexpressed and underexpressed genes were defined by Z-scores, respectively.

### Mouse Tet1 and Tet2 ChIPseq

Tet1 ChIPseq data from mESC (GSM659799) [49] and Tet2 ChIPseq data from mouse bone marrow (GSM897581) [50] were published previously.

### Statistical analyses

All analyses were conducted using the statistical software R (version 3.4.4). The *P* values for boxplots in Figs. 1h, 2b–d, 4j, and 5c were calculated using Wilcoxon rank-sum test and for barplots in Figs. 2a, 3d, 4b, g and h, and 5a were calculated using Welch two sample *t*-test. Odds ratios were calculated using Fisher's exact test in Fig. 4e. *P* values for DNA methylation and gene expression correlation in Fig. 6b were calculated by Pearson correlation. The *P* values of < 0.05 were considered statistically significant for all tests. Pathway enrichment within CCN-CpGs and CIMP-CpG-associated genes was determind using Kyoto Encyclopedia of Genes and Genomes (KEGG, http://www.genome.jp) database. Unique pathways that were below the adjusted *P* value of 0.01 were reported in Fig. 6a. Detailed statistical and bioinformatics analyses are described together with relevant data set.

## Supplementary information

**Additional file 1: Figure S1.** TCGA mRNA RNA-seq data showing TET1, TET2 and hMLH1 expression levels. **Figure S2.** Western blot showing MAFG levels in un-transduced Colo320 and Caco2, ectopically expressing BRAF^V600E^ (*braf*^*V600E*^) or GFP (*gfp*) transduced cells and cell lines constitutively expressing BRAF^V600E^ (Co115 and HT29). **Figure S3.** DNA methylation at mouse *Tet1* and *Tet2* promoter-associated CGI in conditional *Braf*^*-V63*7^ knock-in mice. **Figure S4.** The UCSC browser view showing *Tet1*binding on its own promoter and on *Tet2* promoter. **Table S1.** Quantitative RT-PCR primer sequences. **Table S2.** Pyrosequencing primer sequences.

## Data Availability

The DNA methylation data generated in this study are available through NCBI GEO reprositary https://www.ncbi.nlm.nih.gov/geo/query/acc.cgi?token=ijedgeiwtbmbvyr&acc=GSE98534 under accession numbers GSE98531 (Colon Cancer, paired normal), GES98532 (Colon Cancer Cell lines Caco2, Co115 and SW620), GSE98533 (BRAF^V600E^-transduced stable Caco2 Cells), GSE124915 (BRAF^V600E^-transduced stable Colo320 Cells), GSE108606 (BrafV637 knock-in mice). Publicly available datasets accessed are available in the following: NCBI GEO: CC DNA methylation https://www.ncbi.nlm.nih.gov/geo/query/acc.cgi?acc=GSE25062 [20] NCBI GEO: TA methylation https://www.ncbi.nlm.nih.gov/geo/query/acc.cgi?acc=GSE48684 [22] EMBL-EBI: TA Methylation https://www.ebi.ac.uk/arrayexpress/experiments/E-MTAB-6952/ [21] EMBL-EBI: SSA Methylation https://www.ebi.ac.uk/arrayexpress/experiments/E-MTAB-6952/ [21] NCBI GEO: Cell line methylation https://www.ncbi.nlm.nih.gov/geo/query/acc.cgi?acc=GSE35573 [57] NCBI GEO: CC mRNA expression https://www.ncbi.nlm.nih.gov/geo/query/acc.cgi?acc=GSE25070 [20] NCBI GEO: TET1 ChIP https://www.ncbi.nlm.nih.gov/geo/query/acc.cgi?acc=GSM659799 [49] NCBI GEO: TET2 ChIP https://www.ncbi.nlm.nih.gov/geo/query/acc.cgi?acc=GSM897581 [50]

## Abbreviations

CGI: CpG islands
CIMP: CpG-island methylator phenotype
CIN: Chromosomal instability
CRC: Colorectal cancer
HNM: Healthy normal mucosa
HP: Hyperplastic
MAPK: Mitogen-activated protein kinase
MSI: Microsatellite instability
SSA: Sessile serrated adenomas
TA: Tubular adenomas
WT: Wild-type
