# Supplementary material for: DNA methylation instability by BRAF-mediated TET silencing and lifestyle-exposure divides colon cancer pathways
Source: Clin Epigenetics. 2019 Dec 16;11:196. doi: 10.1186/s13148-019-0791-1 (PMC6916434; doi:10.1186/s13148-019-0791-1)
Supplement: Supplementary file 1 — Additional file 1: Figure S1. TCGA mRNA RNA-seq data showing TET1, TET2 and hMLH1 expression levels. Figure S2. Western blot showing MAFG levels in un-transduced Colo320 and Caco2, ectopically expressing BRAFV600E (brafV600E) or GFP (gfp) transduced cells and cell lines constitutively expressing BRAFV600E (Co115 and HT29). Figure S3. DNA methylation at mouse Tet1 and Tet2 promoter-associated CGI in conditional Braf-V637 knock-in mice. Figure S4. The UCSC browser view showing Tet1binding on its own promoter and on Tet2 promoter. Table S1. Quantitative RT-PCR primer sequences. Table S2. Pyrosequencing primer sequences. [file 13148_2019_791_MOESM1_ESM.docx]

**Additional file 1**

**DNA methylation instability by BRAF-mediated TET silencing and lifestyle-exposure divides colon cancer pathways**

Faiza Noreen^1,2^, Taya Küng^1^, Luigi Tornillo^3^, Hannah Parker^4^, Miguel Silva^5,^ Stefan Weis^1^, Giancarlo Marra^4^, Roland Rad^5^, Kaspar Truninger^1,6*^, Primo Schär^1*^

^1^Department of Biomedicine, University of Basel, Basel, 4058, Switzerland;^2^Swiss Institute of Bioinformatics, Basel, 4053, Switzerland; ^3^Institute of Pathology, University Hospital Basel, Basel, 4056, Switzerland; ^4^Institute of Molecular Cancer Research, University of Zurich, Zurich, 8057, Switzerland; ^5^Department of Medicine II, Klinikum Rechts der Isar, Technische Universität München, 81675 Munich, Germany; ^6^Gastroenterologie Oberaargau, Langenthal, 4900, Switzerland


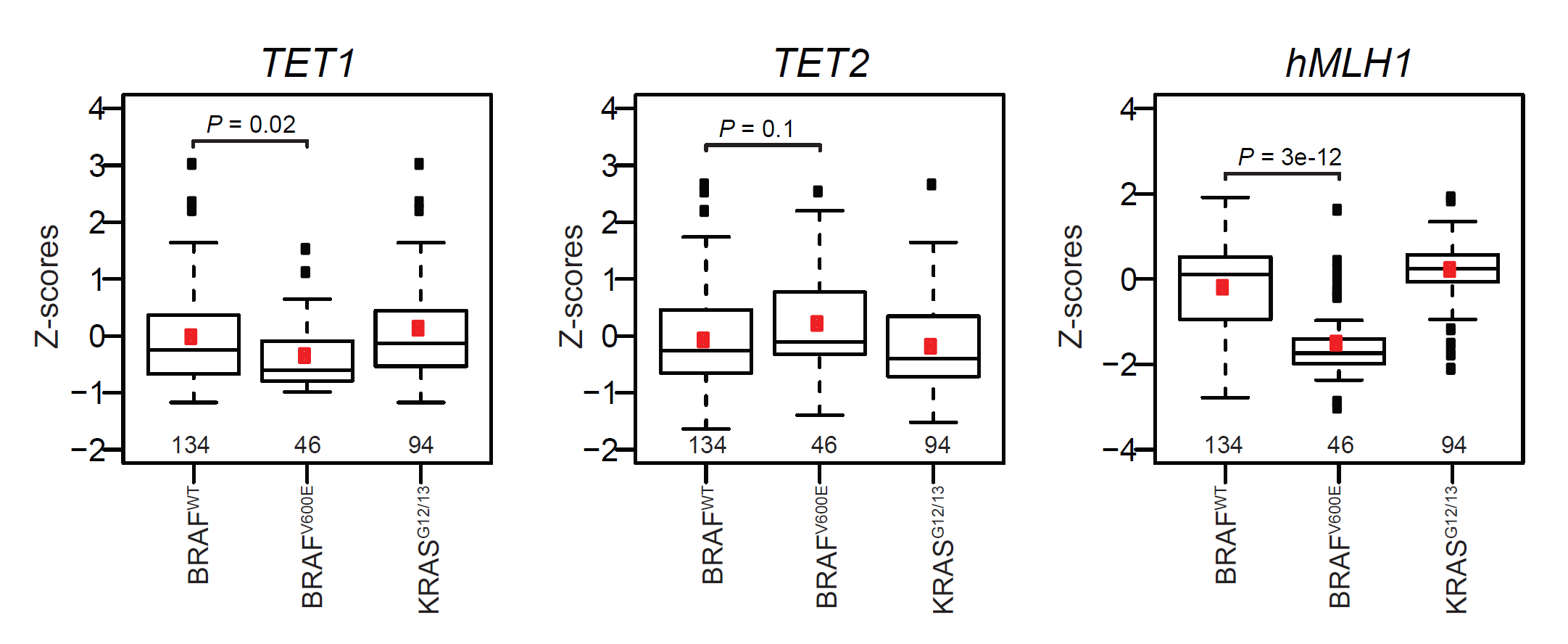


**Figure S1.** TCGA mRNA RNA-seq data showing TET1, TET2 and hMLH1 expression levels. The Z-scores were available for 274 female colon cancer samples (BRAF^WT^/KRAS^WT^; n = 134, BRAF^V600E^; n= 46, KRAS^G12/13^; n=94). The z-scores were calculated using cancer diploid for each gene as the reference population. Number of cancer samples in each category are shown at the bottom of each plot.


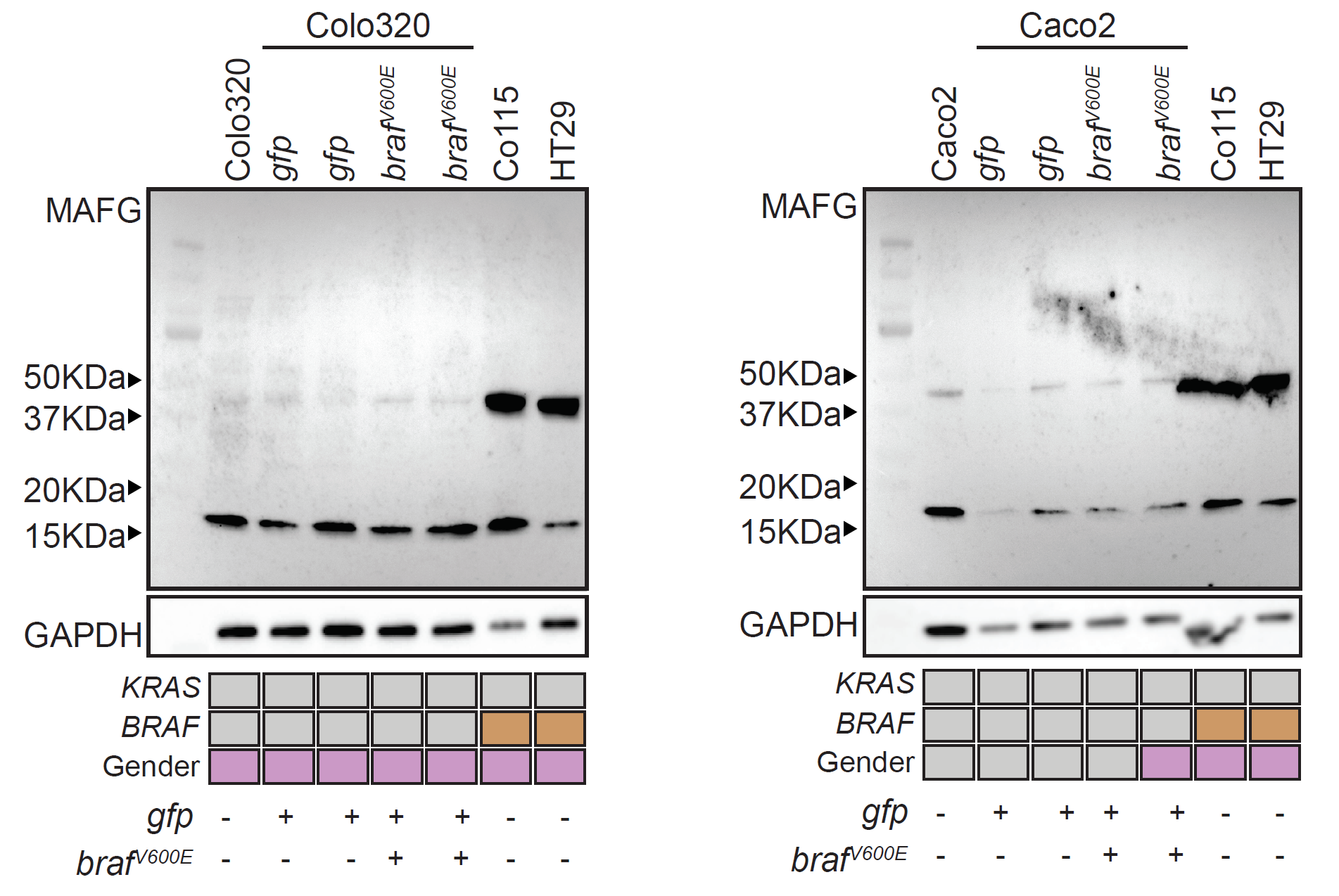


**Figure S2.** Western blot showing MAFG levels in un-transduced Colo320 and Caco2, ectopically expressing BRAF^V600E^ (*braf^V600E^*) or GFP (*gfp*) transduced cells and cell lines constitutively expressing BRAF^V600E^ (Co115 and HT29). In addition to the expected size of the protein (18KDa), the antibody detected a higher band (~42KDa). At both 18 and 42KDa, no increase in MAFG levels was found in *braf^V600E^* versus *gfp*. GAPDH was used as control.


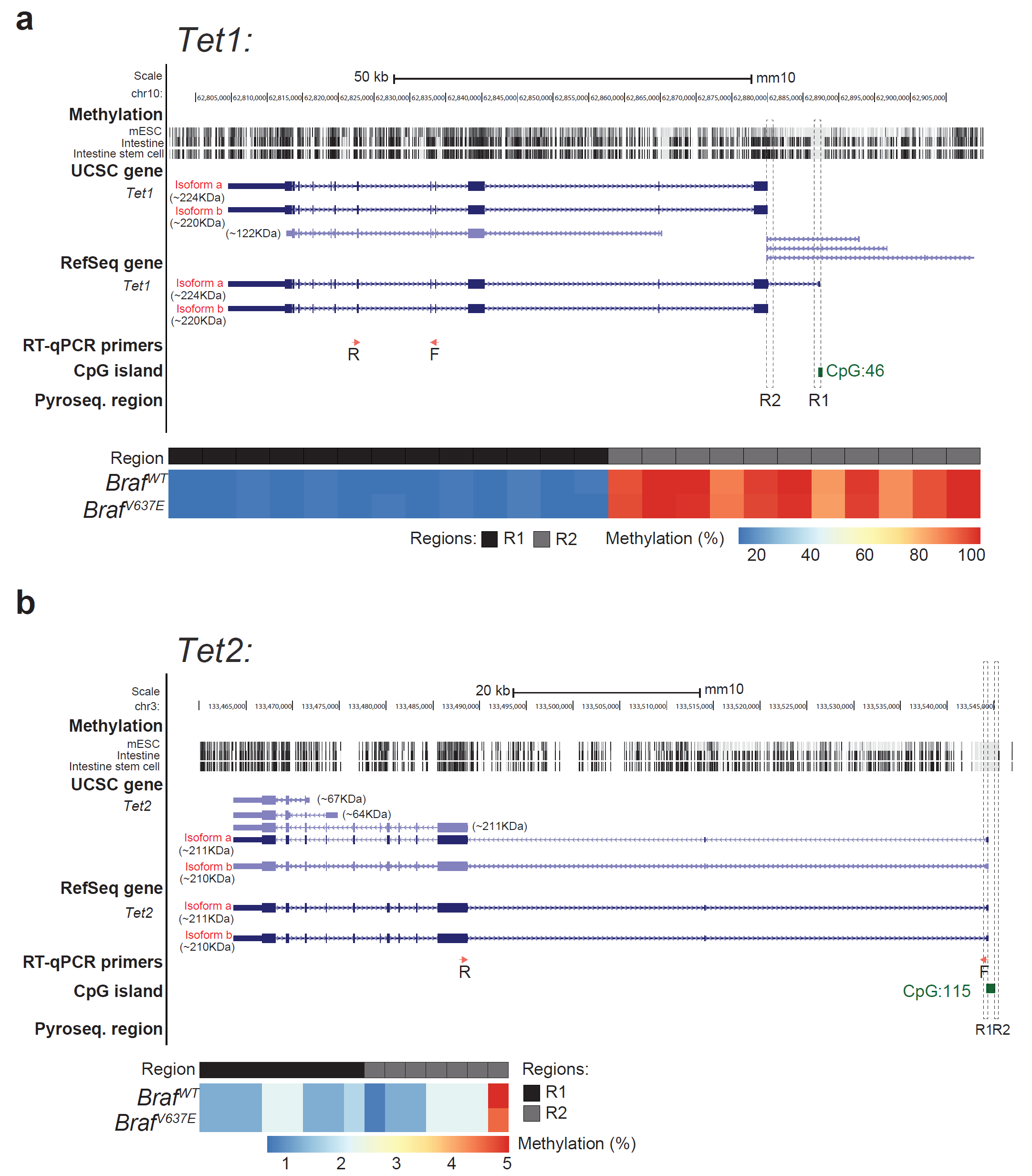


**Figure S3.** DNA methylation at mouse *Tet1* and *Tet2* promoter-associated CGI in conditional *Braf^-V63^*^7^ knock-in mice. (**a**), Snapshot of mouse (mm10) *Tet1* gene in the UCSC browser showing the UCSC and Refseq transcript tracks. In darkblue are the transcript that have been reviewed or validated by either the RefSeq, SwissProt or CCDS staff gene track and in light blue are the other RefSeq transcripts. Location of forward (F) and reverse (R) primers used for RT-qPCR are depicted in red arrows. Region used for the detection of methylation by pyrosequencing (R1and R2) is highlighted with dashed block. Sequence of primers are shown in Supplemental Table 2. Methylation data from mouse embryonic stem cells (mESC), intestine, intestinal stem cell was used from Smith’s lab provided at UCSC browser. Further details of each track can be found at <https://genome-.ucsc.edu>. Below; Heatmaps showing DNA methylation levels (percentage methylation) at *Tet1* region1(R1) and 2 (R2) in intestinal tissue of wild type BRAF (*BRAF^WT^*, n = 5) and mutated knock-in BRAF (*Braf^-V63^*^7^, n = 12) mice. (**b**), Snapshot of mouse (mm10) *Tet2* gene in the UCSC browser. Figure display is the same as in panel a.


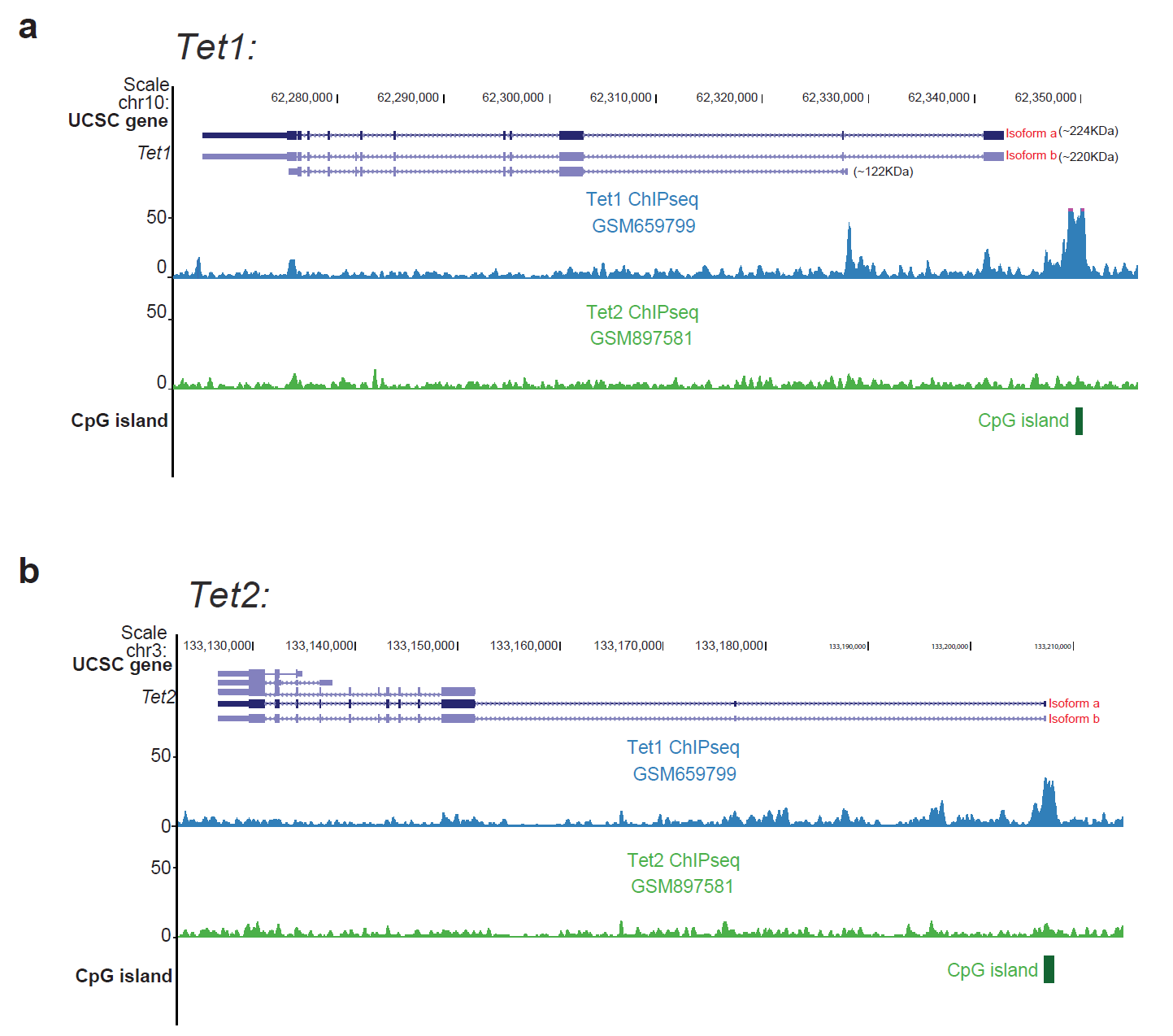


**Figure S4.** The UCSC browser view showing *Tet1* binding on its own promoter and on *Tet2* promoter. Snapshot of mouse (mm9) *Tet1* (**a**) and *Tet2* (**b**) gene in the UCSC browser showing the UCSC and transcript tracks. In darkblue are the transcript that have been reviewed or validated by either the RefSeq, SwissProt or CCDS staff gene track and in light blue are the other transcripts. Tet1 ChIPseq data from mESC (GSM659799) and Tet2 ChIPseq data from mouse bone marrow (GSM897581) are shown in blue and green, respectively.

**Table S1.** Quantitative RT-PCR primer sequences.

| Gene | Forward Primer (5’-3’) | Reverse Primer (5’-3’) |
| --- | --- | --- |
| Human |  |  |
| *BRAF* | TGATTTTGGTCTAGCTACAGT | TGAATAAGGTAACTGTCCAG |
| *BRAFV600E* | TGATTTTGGTCTAGCTACAGA | TGAATAAGGTAACTGTCCAG |
| *TET1* | GCGCGAGTTGGAAAGTTT | GCTCAGTCACACAAGGTTTTGG |
| *TET2* | CCAATAGGACATGATCCAGG | TCTGGATGAGCTCTCTCAGG |
| *hMLH1* | GCTATCAAAGAGATGATTGAGAACTG | TATCCAGATCTTCTTTCCTGATCC |
| *IGFBP7* | CACTGGTGCCCAGGTGTACT | TTGGATGCATGGCACTCATA |
| *NEGR1* | TCATTCCCAGATGTGAGGAAAGTAA | GGTCACGGTGCCAGATTTAATT |
| *FSTL1* | GACCACGATGTGGAAACGCT | AGCTCATCACGGTTGGACTG |
| *FOXE3* | CAGACATGTTCGACAACGGC | CTGTCGACGCTGAACAGAC |
| *SLIT1* | CATTGGTGCCAACCCCCTAT | GTCTCGACCCTTATAGCCGC |
| *FAM78A* | GACTCCAGGGAGAGTCTGCG | GCTGGACATGCCCTGCTC |
| *SLC6A4* | ACGAGCTTCGTTTCGGGATT | GTAGGCCCCTCCAAAAGTCAG |
| *KCNK13* | GTCGTTTCCACCATAGGGTTTG | ATGATGGTGATCAGGCGCTC |
| *RAB31* | CTTCTCGGGGACACTGGGG | CTGCAGCTGAGCCTCGATAG |
| *GREM1* | CACTCTCGGTCCCGCTGA | CTGTGCGGCTCATACTGTCA |
| *DKK3* | CACCCTCAATGAGATGTTCC | ATTTCCAACCTTCGTGTCTG |
| *ACTB* | AGCCTCGCCTTTGCCGA | CTGGTGCCTGGGGCG |
| *GAPDH* | GGAGCGAGATCCCTCCAAAAT | GGCTGTTGTCATACTTCTCATGG |
| *Mouse* |  |  |
| *Tet1* | ACACACCTTGGGGCAGGACCA | TCTGATCACCCACTTGGCGACC |
| *Tet2* | ATATTGATGCGGAGGCGAGG | AATGAATCCAGCAGCACCGT |
| *Mlh1* | TTAGTGAGCGGTGCCATGAG | ACAGCTCTTCACTGAGCTTGG |
| *Actb* | ACTATTGGCAACGAGCGGTT | ACACTTCATGATGGAATTGAATGTAGT |
| *Gapdh* | GTGTTCCTACCCCCAATGTGT | ATTGTCATACCAGGAAATGAGCTT |
| *Tbp* | CACAGGAGCCAAGAGTGAAGAAC | CTACTGAACTGCTGGTGGGTC |

**Table S2.** Pyrosequencing primer sequences.

| Gene | Primer | Sequence (5’-3’) |
| --- | --- | --- |
| Human |  |  |
|  |  |  |
| *TET1* |  |  |
|  | Forward1 | GAGGTTTGTTTTGGGGAGATA |
|  | Reverse1 | GCCCCCGCCCGCCCCAACTCCAAACCTAC |
|  | Forward-nested1 | TGTTTTGGGGAGATATAGTTG |
|  | Sequencing-R1 | GGGGGTTGATTTGG |
|  | Sequencing-R2 | GGGTTTTGATTGTGTTGG |
|  | Forward2 | AGGGTTGGTGTAGGTTTGGAGTT |
|  | Reverse2 | GCCCCCGCCCGACACTTACCCTCCCCCA |
|  | Forward-nested2 | GTAGGTTTGGAGTTGGGGGT |
|  | Sequencing-R3 | TGTTTTGGGAATGTGATT |
|  | Sequencing-R4 | TTGTTTTAGGGGTGGGTT |
|  | Sequencing-R5 | GAGATAAATAATGTTTT |
|  | Sequencing-R6 | TTTGGGGTTTTGATT |
| *TET2* |  |  |
|  | Forward | GAAGTGGTGGTGGAGTGTAG |
|  | Reverse | GCCCCCGCCCGCAATAAAAACCTAAACTACCCTCAC |
|  | Forward-nested | GAGTGTAGATTAGTAAAAAGTTTTAAAGGG |
|  | Sequencing-R1 | GGGAAATTTTAGATGTTA |
|  | Sequencing-R2 | TTTAGGGGTGGAGATT |
|  | Sequencing-R3 | TTAGTTTTGGTTTTTATTT |
| *hMLH1* |  |  |
|  | Forward | TGTATTGGTATATAAAGTTTTTTTTATTTTAGT |
|  | Reverse | GCCCCCGCCCGCTCCTCCTCTCCCCTTA |
|  | Forward-nested | GAGTAGTTTTTTTTTTAGGAGTGAAGG |
|  | Sequencing-R1 | TAGGAGTGAAGGAGGTTA |
|  | Sequencing-R2 | ATAGATTAGGTATAGGGTTTTAT |
|  | Sequencing-R3 | GTTATAAGAGTAGGGTTA |
|  |  |  |
| *Mouse* |  |  |
|  |  |  |
| *mTet1* |  |  |
|  | Forward1 | TGATGTTAAGTTTGGAGGTTTTTAT |
|  | Reverse1 | GCCCCCGCCCGACCCTAAAATCCAAAAACAATTT |
|  | Forward-nested1 | AGGGTTAATTAAATTTTTTGGGGAG |
|  | Sequencing-R1 | GAGTATTGTTAGTTATT |
|  | Forward2 | AAGGAGTTTGGGTTTTGAAAAAG |
|  | Reverse2 | GCCCCCGCCCGCATCCTTACATCTTTACTTACTCTACA |
|  | Forward-nested2 | TTTGGGTTTTGAAAAAGAATTTGG |
|  | Sequencing-R2 | TTGAAAAAGAATTTGGTTT |
|  |  |  |
| *mTet2* |  |  |
|  | Forward1 | GGGAGATTGGAAAGGGATTAT |
|  | Reverse1 | GCCCCCGCCCGAAAACAAAATAACTACCCTATAAAATT |
|  | Forward-nested1 | GAGGAAAGAAAGAGAGAGAGAG |
|  | Sequencing-R1 | AGAGAGAGAGAGATAGG |
|  | Forward2 | GGGAGAGTTAGAGGGGTTTGG |
|  | Reverse2 | GCCCCCGCCCGCTTAAACATTAAAAACTACTAACTTAATTC |
|  | Forward-nested2 | GTTTAGGTTTATTTTTTGTTTATTTTGG |
|  | Sequencing-R2 | ATTTTTTGTTTATTTTGGTTA |
|  |  |  |
| Universal primer |  | Biotin-GCCCCCGCCCG |

Sequence underlined represents the 11-base tag.
